# OR13-004 – Evidence-based clinical classification criteria for periodic fevers

**DOI:** 10.1186/1546-0096-11-S1-A266

**Published:** 2013-11-08

**Authors:** S Federici, S Ozen, I Koné-Paut, H Lachmann, P Woo, L Cantarini, G Amaryan, A Insalaco, J Kuemmerle-Deschner, B Neven, N Dewarrat, Y Uziel, D Rigante, T Herlin, S Martino, A Simon, S Stojanov, H Ozdogan, J Frenkel, N Ruperto, A Martini, M Sormani, M Hofer, M Gattorno

**Affiliations:** 1 GASLINI, Genova, Italy

## Introduction

No evidence-based classification criteria are so far available the majority of autoinflammatory diseases

## Objectives

To elaborate and validate a set of clinical criteria able to correctly classify patients affected with the most common periodic fevers

## Methods

All FMF, TRAPS, MKD and CAPS patients enrolled in the Eurofever registry until March 2013 were evaluated. For each disease gold standards were considered according to the following criteria: i) clinical validation by centers and disease-principal investigator, ii) confirmative molecular analysis (2 mutations for MEFV with at least one mutation in exon 10, 2 mutations of MVK gene, 1 mutation of TNFRFS1A with exclusion of low-penetrance variants, 1 mutation of NLRP3 with exclusion of low-penetrance variants), iii) PFAPA patients validated by disease-principal investigator and confirmed by the centers on the basis of the follow-up. Clinical criteria was formulated on the basis of a univariate and multivariate analysis in a first group of patients (training set) and validated in an independent set of patients (validation set).

## Results

A total of 1204 consecutive patients with periodic fevers were enrolled in the registry. Among them 743 consecutive gold standard patients (288 FMF, 73 MKD, 96 TRAPS, 87 CAPS, 199 PFAPA) were evaluated (440 in the training set and 303 in the validation set).The multivariate analysis identified the clinical variables (either as presence or absence).

The classification score was than tested in an independent set of patients (validation set) revealing a sensitivity of 93% and specificity of 89% for FMF; a sensitivity of 100% and specificity of 74% for TRAPS; a sensitivity of 80% and specificity of 90% for MKD and sensitivity of 97% and specificity of 92% for CAPS; sensitivity of 99% and specificity of 96% for PFAPA. The performance in non-gold standard patients (i.e. heterozygous patients in autosomal recessive diseases or patients with low-penetrance mutations) revealed a variable percentage of patients (70% FMF, 75% TRAPS, 41% MKD and 94% CAPS) positive for the respective criteria.

independently correlated to for each disease with their specific weight. The cut off value of the classification score was chosen on the ROC curve in order to guarantee the highest sensitivity and specificity.

## Conclusion

Evidence-based clinical criteria for the classification of patients with inherited periodic fevers have been elaborated. These clinical criteria could be used in association with molecular analysis and other variables (i.e. metabolic examinations, response to specific treatments) for patients classification.

## Disclosure of interest

None declared

